# Alzheimer’s disease and epilepsy: An increasingly recognized comorbidity

**DOI:** 10.3389/fnagi.2022.940515

**Published:** 2022-11-10

**Authors:** Fei Yang, Liling Chen, Yanying Yu, Tingwan Xu, Lu Chen, Wenqian Yang, Qian Wu, Yanbing Han

**Affiliations:** Department of Neurology, First Affiliated Hospital of Kunming Medical University, Kunming, China

**Keywords:** Alzheimer’s disease, epilepsy, comorbidity, seizure, cognition

## Abstract

Both Alzheimer’s disease (AD) and epilepsy are common chronic diseases in older people. Seizures and epileptiform discharges are very prevalent in AD and can occur since any stage of AD. Increasing evidence indicates that AD and epilepsy may be comorbid. Several factors may be related to the underlying mechanism of the comorbidity. Identifying seizures in patients with AD is a challenge because seizures are often clinically non-motor and may overlap with some AD symptoms. Not only seizures but also epileptiform discharges may exacerbate the cognitive decline in AD patients, highlighting the importance of early recognition and treatment. This review provides a comprehensive overview of seizures in AD from multiple aspects to provide more insight.

## Introduction

Alzheimer’s disease (AD) is the most common cause of dementia. The AD patient population is rapidly expanding to 50 million people worldwide (Scearce-Levie et al., 2020). Most cases occur after age 65 and are referred to as late-onset AD (LOAD), while cases earlier than age 65 are much rarer, accounting for less than 5%, and are referred to as early-onset AD (EOAD) (Long and Holtzman, 2019). With the aging population, it is estimated that the incidence of AD will continue to increase, which will bring a heavy burden to society and families of AD patients (Teixeira et al., 2017; Jia et al., 2020; Scheltens et al., 2021).

Epilepsy is characterized by spontaneous seizures. Globally, epilepsy affects more than 70 million people, with the highest incidence in patients older than 65 years (Thijs et al., 2019). Neurocognitive disorders, including AD, are a common cause of new-onset epilepsy in older people. Recurrent seizures may be followed by cognitive decline, particularly in older patients (Sen et al., 2020; Wang et al., 2021).

Both AD and epilepsy have a serious impact on the patients’ health and reduce the quality of life of the patient and their family. Unfortunately, both can occur in one patient at the same time. Since Sjogren et al. (1952) reported that seizures were confirmed in patients with AD by pathological diagnosis, the relationship between AD and epilepsy has attracted an increasing amount of attention. Research on the relationship between seizures and AD has developed quickly.

## High incidence of seizures and epileptiform discharges in Alzheimer’s disease

Table 1 lists the prevalence of seizures or epileptiform discharges in AD patients reported in various studies since 1952 (Sjogren et al., 1952; Hauser et al., 1986; Mendez et al., 1994; Mendez and Lim, 2003; Amatniek et al., 2006; Hommet et al., 2007; Bell et al., 2011; Vossel et al., 2013; Horváth et al., 2016; Nicastro et al., 2016; Vossel et al., 2016; Zarea et al., 2016; Beagle et al., 2017; Horváth et al., 2018; Rauramaa et al., 2018; Lam et al., 2020). These results are inconsistent and may be related to diverse study subjects, objectives, and strategies. The incidence of seizures in AD patients is much higher than that in the general population. Patients with AD were 5 to 10 times more likely to develop seizures than those without AD at the same age (Hauser et al., 1986; Larner, 2010; Corbett et al., 2017). During the course of AD, 10 to 22% of patients had at least one unprovoked seizure. Previously, it was generally believed that the first seizure occurred in the middle and late stages of AD, and the incidence increased with the progression of the disease (Hauser et al., 1986; Hesdorffer et al., 1996; Mendez and Lim, 2003; Scarmeas et al., 2009). The cumulative risk of seizures increased from 11 to 26% between 10 and 15 years after the diagnosis of AD (Mendez and Lim, 2003). In addition, the prevalence of seizures was higher in EOAD than in LOAD. People with EOAD were nearly twice as likely to have seizures (11%) as people with LOAD (6%) (Sulkava, 1982). In a large French cohort of autosomal dominant EOAD (ADEOAD), 47.7% of the patients suffered from at least one seizure during a mean follow-up of 8.4 years (Zarea et al., 2016). In fact, except for clinical seizures, electrophysiological (EEG) monitoring often shows subclinical and interictal epileptiform discharges in AD patients. Increasing evidence supports the emergence of epileptiform discharges in early AD (Vossel et al., 2013; Lam et al., 2020). Vossel et al. (2016) observed that up to 42.4% of AD patients had subclinical epileptiform activity, which was defined as paroxysmal sharp waveforms lasting 20 to 200 ms and disrupting background activity (Vossel et al., 2017), using overnight long-term monitoring with video-EEG (LTM-VEEG) or magnetoencephalography with simultaneous EEG (M/EEG). Thus, the incidence of epileptiform discharges in AD may be much higher than that of clinically noticeable seizures. How to identify seizures and epileptiform discharges early and understand the true incidence in AD deserve further attention. In order to evaluate AD patients effectively, clinicians and researchers should conduct sensitive and comprehensive neurophysiological assessments.

**Table 1:** Studies investigating seizures/ epileptiform discharges incidence in patients with Alzheimer’s disease.

Authors	Study type	Study population	Number of samples	Age mean (SD)	Seizures/Epileptiform discharges incidence	Events confirmed type
Sjogren et al. (1952)	Retrospective	AD (autopsy)	18	53.0 ± 5.0	22% (4/18)	Clinical
Hauser et al. (1986)	Retrospective	AD (autopsy)	81	–	20% (16/81)	Clinical
Mendez et al. (1994)	Retrospective	AD (autopsy)	446	64.1 ± 8.8	17% (77/446)	Clinical
Mendez and Lim (2003)	Systematic review	AD	874	–	10–20%	–
Amatniek et al. (2006)	Prospective	Probable AD	236	–	7.7% (12/236)	Clinical or EEG
Hommet et al. (2007)	Prospective	Clinical AD	197	≥65	2.5% (5/197)	Clinical
Bell et al. (2011)	Prospective	Clinical AD	28,089	42–101	2.1%	–
Vossel et al. (2013)	Retrospective	aMCI and probable AD	1,257	68.0 ± 7.8 (aMCI-Epilepsy)	4.3% (54/1257)	Clinical or EEG or LTM-EEG
				69.1 ± 9.0 (AD-Epilepsy)		
Nicastro et al. (2016)	Systematic review	AD	3,555,817	–	0.5–64%	–
Horváth et al. (2016)	Systematic review	AD	3,506,623	–	0.5–64%	–
Vossel et al. (2016)	Prospective	Probable AD	33	61.7 ± 67.4	42.4% (14/33)	LTM-EEG or M/EEG
Zarea et al. (2016)	Prospective	ADEOAD	132	44.8 (24–63)	47.7% (63/132)	Clinical
Beagle et al. (2017)	Retrospective	Probable AD	1,320	62–78	13.4% (177/1320)	Clinical or EEG
Rauramaa et al. (2018)	Retrospective	AD (autopsy)	64	81.4 ± 8 (AD-Epilepsy)	17% (11/64)	Clinical
Horváth et al. (2018)	Cross-sectional	Probable AD	42	68.5 ± 4.1 (AD-Seizures) 68.6 ± 10.9 (AD-Epileptiform discharges)	52% (22/42)	Ambulatory EEG
Lam et al. (2020)	Cross-sectional	Probable AD-NoEp	41	76.3 ± 7.2	22% (9/41)	Ambulatory EEG

AD, Alzheimer’s disease; aMCI, amnestic mild cognitive impairment; ADEOAD, autosomal dominant early-onset Alzheimer’s disease; AD-NoEp, probable AD with no history/risk factors for epilepsy; EEG, electroencephalogram; LTM-EEG, long-term monitoring with video-EEG; M/EEG, magnetoencephalography with simultaneous EEG.

## Risk factors for seizures in Alzheimer’s disease

Potential risk factors for seizures in AD patients include onset age of dementia, down syndrome (DS), epileptiform discharges on EEG, sharp decline in cognition, and medication (Spencer, 2014; Asadollahi et al., 2019). A study based on a representative database from the Nationwide Inpatient Sample among the elderly population older than 55 years revealed that the younger AD patients were, the more likely they were to have seizures (Sherzai et al., 2014). People with EOAD were nearly twice as likely to have seizures (11%) as people with LOAD (6%) (Sulkava, 1982). Seizures were also a common feature in patients with ADEOAD (Zarea et al., 2016). DS patients above 45 years were more likely to develop AD, and it was found that up to 84 percent of these patients suffered from seizures (Menéndez, 2005). Another study showed that patients with DS dementia were more likely to have seizures earlier (Gholipour et al., 2017). Researchers have suggested that epileptiform discharges might be a predictive factor of clinical seizures (Vossel et al., 2013; Asadollahi et al., 2019). Both seizures and epileptiform discharges might impair cognitive function and lead to a sharp decline in cognitive function in patients with AD (Volicer et al., 1995; Vossel et al., 2016). In turn, when AD patients experience sudden cognitive decline, they should be alert to the presence of seizures or epileptiform discharges. Moreover, some medications used in AD patients may decrease the seizure threshold. Antipsychotic drugs, such as clozapine and quetiapine, were found to increase the risk of seizures (Kumlien and Lundberg, 2010). Many studies have reported epileptic seizures in AD patients treated with these drugs (Wong and Delva, 2007; Shao et al., 2016). Therefore, patients with AD should be aware of seizures, particularly when they are using antipsychotic drugs. In addition, patients with AD may suffer from stroke, traumatic brain injury, encephalitis, metabolism and toxic damage, which are common risk factors that induce seizures in the general population (Beghi et al., 2010; Cretin et al., 2017).

## Potential mechanisms for comorbidity of Alzheimer’s disease and epillepsy

**Figure 1:**
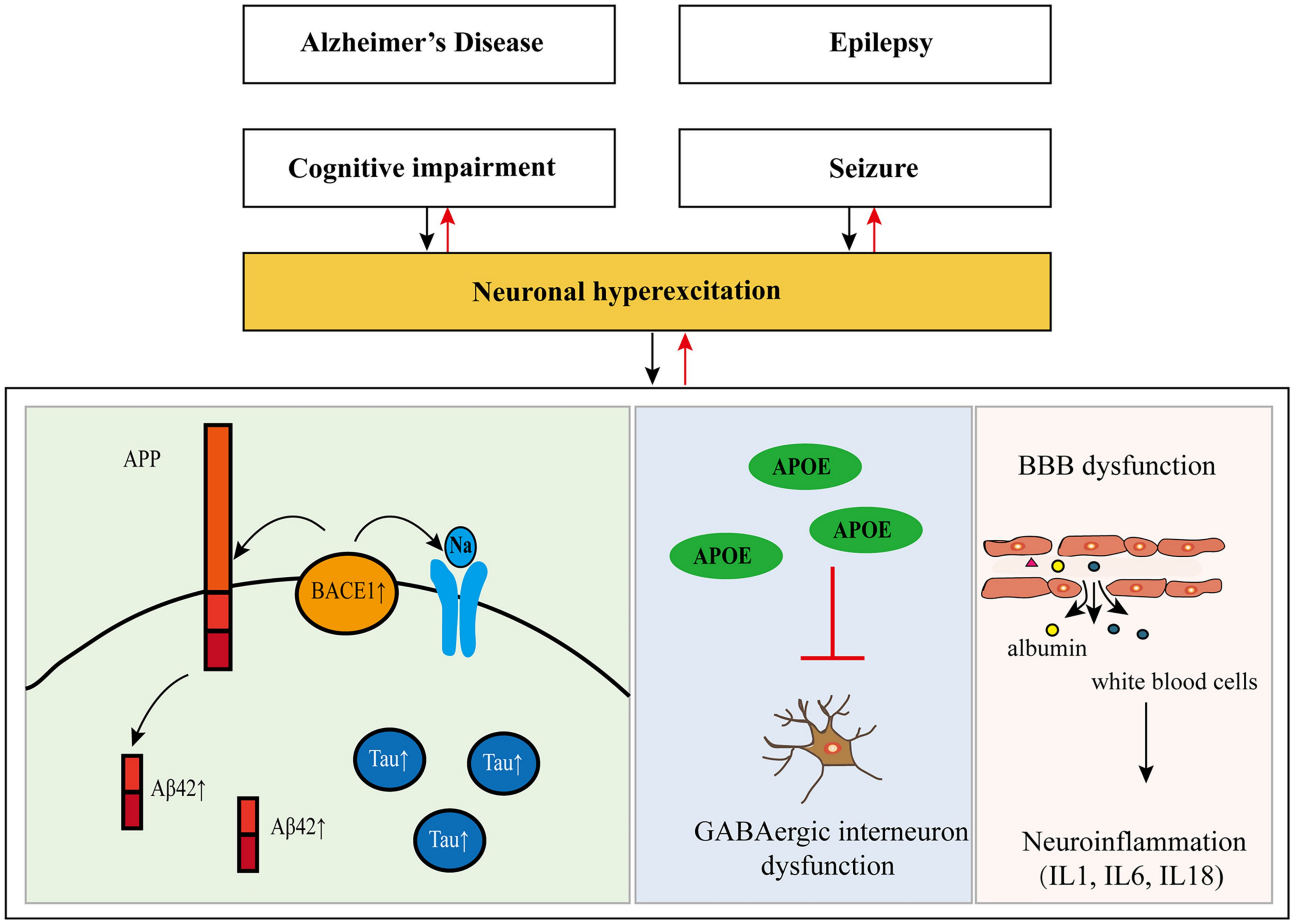
Pathogenesis of Alzheimer’s disease and epilepsy and their relationship. APP, amyloid precursor protein; Aβ, amyloid-β; Tau, protein tau; BACE1, β-site amyloid precursor protein cleaving enzyme 1; APOE, apolipoprotein E; GABA, gamma-aminobutyric acid; BBB, blood–brain barrier.

In recent years, some researchers have found that there is a two-way relationship between epilepsy and dementia, especially AD (Giorgi et al., 2020; Stefanidou et al., 2020). Hippocampal atrophy is present in almost all patients with AD, while hippocampal sclerosis (HS) is also a frequent pathological change in epileptic patients, suggesting that AD and epilepsy may have a common anatomical basis. Furthermore, many elements involved in the pathogenesis of AD have been found to regulate neuronal excitability, and seizures result from excessive neuronal excitability (see Figure 1 and Table 2).

**Table 2:** Clinical and preclinical studies of elements involved in the pathogenesis of AD and epilepsy.

Elements	Study type	Sample type	Findings	Authors
Aβ	Clinical study	Patients with drug-resistant TLE	Full-length APP expression in hippocampus (172% of controls, *p* < 0.01); phosphorylated APP (Thr668) increased levels in both hippocampus (249% of controls, *p* < 0.01) and temporal cortex (237% of controls, *p* < 0.0001); Aβ_1-42_ was upregulated in the hippocampus (239% of controls, *p* < 0.01)	Gourmaud et al. (2020)
Aβ	Clinical study	Patients with late-onset epilepsy	Significant (37.5%) prevalence of CSF pathological Aβ_1-42_, 7.5% of which displayed an AD-like CSF pattern	Costa et al. (2019)
Aβ	Preclinical study	Epileptic rat model of kainic acid	Increased levels/processing of APP, enhanced production of Aβ-related peptides	Kodam et al. (2019)
Aβ	Preclinical study	APP/PS1 transgenic mice	Overexpression of Aβ altered the neuronal discharge	Tamagnini et al. (2015)
Aβ	Preclinical study	AD model of hAPP transgenic mice	Stimulation of neuronal activity facilitates Aβ release	Rodriguez et al. (2020)
Aβ	Preclinical study	AD model of APP transgenic mice	Increased Aβ level was associated with overfiring of neurons and circuits	Harris et al. (2020)
Tau	Clinical study	Patients with drug-resistant TLE	Thirty-one of 33 cases (94%) of tau hyperphosphorylation	Tai et al. (2016)
Tau	Clinical study	Patients with drug-resistant TLE	Tau protein in the hippocampus (297% of controls, *p* < 0.01) and its pathological phosphorylation (198% of controls, *p* < 0.05)) were increased	Gourmaud et al. (2020)
Tau	Clinical study	Patients with AD	Seizures was associated with CSF tau (hazard ratio = 1.001; 95% confidence interval (CI) = [1.001,1.002]; *p* = 0.001)	Tábuas-Pereira et al. (2019)
Tau	Preclinical study	Rat model of TLE	Tau in parahippocampal cortex tissue during the latency phase	von Rüden et al. (2020)
Tau	Preclinical study	Primary cortical neuronal cultures	Neuronal activity stimulates tau release from neurons	Pooler et al. (2013)
Tau	Preclinical study	Human tau transgenic mice	Significant excitotoxicity	Decker et al. (2016)
BACE1	Clinical study	Patients with epilepsy	Significant correlation (r = 0.684, *p* < 0.0001) between the expression level of BACE1 and its natural antisense	Mazdeh et al. (2018)
BACE1	Clinical study	Patients with drug-resistant TLE	Expression of BACE1 was increased (159% of controls, *p* < 0.05 in the hippocampus and 189% of controls *p* < 0.001 in the temporal cortex)	Gourmaud et al. (2020)
BACE1	Preclinical study	BACE1-deficient mice	BACE1-mediated pathological changes are related to abnormal sodium channel function	Lehnert et al. (2016)
BACE1	Preclinical study	BACE1-deficient mice	BACE1 deficiency causes altered neuronal activity and neurodegeneration	Hu et al. (2010)
BBB dysfunction	Clinical and preclinical study	Patients with AD and epilepsy Animal model AD and of epilepsy	AD with diffuse and focal (epilepsy) BBB dysfunction Paroxysmal slow cortical activity is associated with BBB dysfunction	Milikovsky et al. (2019)
BBB dysfunction	Preclinical study	Endothelial Cdk5 deficit mice	Seizures lead to BBB dysfunction and related changes	Liu et al. (2020)
Neuroinflammation	Clinical and preclinical study	Patients with epilepsy, animal models of epilepsy	Neuroinflammatory signaling plays an important role	Hodges and Lugo (2020)
Neuroinflammation	Preclinical study	AD model mice	AD pathogenesis interactions with immunological mechanisms	Heneka et al. (2015)
Neuroinflammation	Preclinical study	APP/PS1 and interleukin-18 deficient mice	Interleukin 18-deficient AD mice develop fatal seizures due to increased neuronal network transmission	Tzeng et al. (2018)
APOE	Clinical study	Patients with TLE	Thirty-four (25%) of 138 patients had tests cores indicating verbal learning deficit (VLD); The presence of an APOE allele was associated with an increased risk of VLD (OR, 4.18; 95%CI, 1.66–10.55)	Gambardella et al. (2005)
APOE	Clinical study	Patients with TLE	APOE4 carriers had epilepsy onset almost 4 years earlier than non-carriers (mean difference 5.15 years; 95%CI 2.08–6.22; *p* = 0.001)	Kauffman et al. (2010)
APOE	Preclinical study	AD model mice	APOE4-mediated GABAergic interneuron loss can lead to inhibitory network deficits	Najm et al. (2019)
GABAergic dysfunction	Clinical study	Patients with AD	Postsynaptic excitatory to inhibitory (E/I) imbalance correlated with GABAergic dysfunction (*R*^2^ = 0.93, *p* < 0.0001)	Lauterborn et al. (2021)
GABAergic dysfunction	Preclinical study	APP/PS1 transgenic mice	Significant (50–60%) decrease in GABAergic neurons	Ramos et al. (2006)
GABAergic dysfunction	Preclinical study	Mice	High correlation (*R*^2^ = 0.74 dashed line) exists between global synaptic E/I ratio and neuronal firing	Iascone et al. (2020)

Aβ, amyloid-β; Tau, protein tau; APOE, apolipoprotein E; BBB, blood–brain barrier; BACE1, β-site amyloid precursor protein cleaving enzyme 1; GABA, gamma-aminobutyric acid; TLE, temporal lobe epilepsy; AD, Alzheimer’s disease; APP, amyloid precursor protein; CSF, cerebrospinal fluid.

Amyloid-β (Aβ) may contribute to seizures in AD by causing excitatory changes in neural networks. One of the pathological features of AD is the presence of extracellular Aβ, also known as Aβ plaques, which are clipped by human amyloid precursor protein (APP). A recent study showed that the expression and phosphorylation of APP and Aβ_1-42_ were upregulated in the brain tissues of patients with drug-resistant temporal lobe epilepsy (TLE). It was also found that the phosphorylation of APP in the hippocampus was inversely correlated with cognitive function (Gourmaud et al., 2020). Patients with late-onset epilepsy of unknown origin (LOEU) had a significant prevalence of cerebrospinal fluid (CSF) pathological Aβ_1-42_, 7.5% of which displayed an AD-like CSF pattern. Moreover, 17.5% of LOEU patients eventually progressed to AD. There was a hazard ratio of 3.4 for those patients with pathological Aβ_1-42_ converted to AD at further follow-up (Costa et al., 2019). In addition, some researchers suggested that Aβ might play a dual role in late-onset epilepsy and AD (Costa et al., 2018; Sen et al., 2018). These findings suggest that epilepsy and AD share the same pathological changes, providing a pathological basis for the comorbidity between the two.

In preclinical studies, APP/PS1 transgenic mice are commonly constructed as AD model mice, which can cause the overexpression of APP in the hippocampus and increase the expression of Aβ. After systemic injection of kainic acid, adult rats suffered from seizures, gliosis, and loss of hippocampal neurons, along with increased levels/processing of APP. The increased APP resulted in the enhanced production of Aβ-related peptides in the epileptic rats, which suggested that APP/Aβ peptides derived from astrocytes might have a role in epileptogenesis (Kodam et al., 2019). The upregulated Aβ level was closely related to abnormal epileptic activity and increased intrinsic excitability of CA1 hippocampal neurons. Moreover, overexpression of Aβ altered the neuronal discharge, action potential waveform, and capacitance of CA1 neurons in aged AD model mice (Tamagnini et al., 2015). Gureviciene et al. (2019) similarly identified several types of epileptic spikes in free-moving APP/PS1 transgenic mice using multiple cortical and subcortical electrodes. Elevated Aβ levels and non-motor seizures were also found in the cortical and hippocampal networks (Palop et al., 2007). Interestingly, stimulation of neuronal activity facilitated Aβ release from neurons *in vivo* (Rodriguez et al., 2020). Additionally, increased neuronal activity enhanced the production of Aβ from APP (Saito et al., 2012). The level of Aβ in hippocampal interstitial fluid (ISF) increased rapidly in APP transgenic mice, while tetrodotoxin (TTX) blocked neuronal activity and thus reduced the level of Aβ (Cirrito et al., 2005). A recent study also confirmed that the number of Aβ plaques was associated with the frequency of epileptiform discharges (Reyes-Marin and Nuñez, 2017). Moreover, the increased Aβ level was associated with hyperexcitability of neurons and circuits (Harris et al., 2020). Thus, we can see that Aβ may be involved in seizures in AD by regulating the excitability of neural networks.

Another key histopathological hallmark of AD is the neurofibrillary tangles of protein tau. Similar to Aβ, tau has also been found in pathological changes in patients with epilepsy. Tai et al. (2016) first performed pathological examination on tissue from 50-year-old to 65-year-old patients with drug-resistant TLE after surgery. They found that 94% of cases showed hyperphosphorylated tau pathology, which was one of the pathological characteristics of AD. Further analysis showed that modified tau scores were strongly negatively correlated with changes in cognitive test scores over 1 year (before and after temporal lobe resection). In a recent study, researchers reconfirmed that tau protein and its pathological phosphorylation were increased in patients with resistant TLE hippocampi, and both were inversely correlated with cognitive function (Gourmaud et al., 2020). Some scholars found that seizure risk in patients with AD was associated with high levels of protein tau in CSF and lower baseline Mini-Mental State Examination (MMSE) scores. They speculated that tau protein might also play a role in regulating neuronal excitability (Tábuas-Pereira et al., 2019). Another study also summarized the linking role of protein tau in AD and epilepsy. Researchers found that hyperphosphorylated tau was associated with the pathogenesis of epilepsy and AD, as well as with cognitive deficits. More interestingly, inhibition of tau not only minimized seizure and AD-like pathology but also improved cognitive decline in both epilepsy and AD (Paudel et al., 2019). These studies provide molecular and pathological evidence for the involvement of tau in AD and epilepsy.

In addition, seizures result from excessive neuronal excitability. Tau may also be involved in the formation of hyperexcitability in neurons. Proteomic analysis revealed the regulation of microtubule-associated tau in parahippocampal cortex tissue during the latency phase in a rat model of TLE (von Rüden et al., 2020). The stimulation of neuronal activity facilitated tau release both *in vitro* (Pooler et al., 2013) and *in vivo* (Yamada et al., 2014). Alpha-amino-3-hydroxy-5-methyl-4-isoxazolepropionic acid (AMPA)-mediated tau release was significantly prevented when tetanus toxin blocked presynaptic vesicle release and when TTX inhibited neuronal activity (Pooler et al., 2013). Expressing human full-length Tau with the Tau mutation in transgenic mice showed significant excitotoxicity caused by dysfunction of increasing extracellular glutamate in hippocampal tissue slices. Significant axonal sprouting of mossy fibers and epileptiform discharges were also found in hippocampal slices (Decker et al., 2016). The sprouting of mossy fibers was shown to play a critical role in epileptogenesis, and tau was also related to the abnormal sprouting of mossy fibers in the pentylenetetrazol (PTZ) kindling model of epilepsy (Tian et al., 2010). Moreover, the severity and frequency of seizures were reduced in the PTZ kindling model of epilepsy after inhibition of protein tau production, which suggested that the reduction of protein tau prevented excitatory toxicity (Roberson et al., 2007). The increase in protein tau changes neuronal excitability and promotes seizure occurrence and the decline in cognitive function in patients with AD, while the decrease in protein tau can improve the cognitive function of epilepsy and AD. These findings may provide a new idea for the treatment of seizures in patients with AD.

β-Site amyloid precursor protein cleaving enzyme 1 (BACE1) is very active in patients and animal models of AD. Aβ production is mediated by continuous cleavage of APP, and the extracellular domain of APP is mediated by BACE1. Therefore, BACE1 is necessary for the production of Aβ (Cole and Vassar, 2007; Ehlers, 2018). In a clinical study, researchers suggested that uncontrolled seizures increased tau, APP, and BACE1 protein levels, which accelerated Aβ_42_ production and tau pathology. They also found cognitive impairment and neuronal dysfunction in drug-resistant epilepsy (Gourmaud et al., 2020). There was a significant correlation between the expression level of BACE1 and its natural antisense (BACE1-AS) in the blood of patients with epilepsy (Mazdeh et al., 2018). In a preclinical study, evidence showed that BACE1-mediated pathological changes were related to abnormal sodium channel function (Kim et al., 2011; Lehnert et al., 2016). Abnormal sodium channels can cause abnormal firing of neurons and lead to seizures. The link between BACE1 and seizures was also demonstrated in animal studies (Hu et al., 2010). Although clinical trials of BACE1 inhibitors conducted to date have been discontinued due to inefficacy or safety reasons, BACE1 remains an effective therapeutic target for AD (Hampel et al., 2021), and researchers should examine whether BACE1 intervention can play a role in seizures in AD patients.

Blood–brain barrier (BBB) dysfunction may be another important link between AD and seizures. The results from patients and animal models of AD suggested that microvascular lesions, especially BBB dysfunction, played an important role (Sweeney et al., 2018). Recently, some scholars found that paroxysmal slow wave events (PSWEs) in patients with AD were recorded as bilateral, while PSWEs were usually focal and colocalized with BBB dysfunction in patients with epilepsy. They identified PSWEs as an EEG manifestation of non-motor seizures in patients with AD and suggested BBB dysfunction pathology. EEG recordings from animal models of AD with diffuse and focal (epilepsy) BBB dysfunction confirmed the occurrence of PSWEs (Milikovsky et al., 2019). Induced seizures, especially status epilepticus, can quickly lead to BBB dysfunction and related changes (Ndode-Ekane et al., 2010; Liu et al., 2020). BBB dysfunction also caused albumin extravasation and white blood cells from the blood into the brain parenchyma, which altered neuronal excitability and induced or promoted the occurrence of seizures (Marchi et al., 2016; Loscher and Friedman, 2020). BBB dysfunction may lead to neuronal hyperexcitability involved in seizures in AD, which could be an underlying mechanism and a promising therapeutic target.

Neuroinflammation may play a role in seizures in patients with AD. Inflammation is a fundamental process in the development of epilepsy and continues to be susceptible to seizures. Neuroinflammatory signaling plays an important role in the development of epilepsy, both in patients with epilepsy and in animal models of acquired epilepsy and inherited epilepsy (Hodges and Lugo, 2020). Inflammatory markers were found in AD model mice (Choi et al., 2013). Increasing evidence suggests that AD pathogenesis is not only restricted to the neuronal compartment but also includes strong interactions with immunological mechanisms in the brain (Heneka et al., 2015). Recently, it was found that APP/PS1 mice deficient in the inflammatory body-derived cytokine interleukin-18 (IL18) showed a lower chemically induced epileptic threshold and a selective increase in gene expression associated with increased neuronal activity. It was suggested that IL18-deficient AD mice developed fatal seizures due to increased neuronal network transmission (Tzeng et al., 2018). These results suggest that neuroinflammation may indeed be involved in the mechanism of epileptic seizures in AD. In general, both AD and epilepsy cause abnormal neuroinflammation. Neuroinflammation, in turn, is a risk factor for seizures and AD progression.

The association between apolipoprotein E (APOE) and AD has been confirmed by many studies (Shi and Holtzman, 2018; Nguyen et al., 2020), while APOE is associated with neural network excitability. An alteration in cognitive performance as a function of the presence of the APOE4 allele was found in TLE patients (Gambardella et al., 2005). In addition, a study investigated the role of APOE4 as a moderator of age at TLE onset. It was confirmed that the onset of epilepsy occurred 4 years earlier in APOE4 carriers than in non-carriers (Kauffman et al., 2010). Gamma-aminobutyric acid (GABA)-ergic interneurons were selectively vulnerable to intracellularly produced APOE4 through a tau-dependent mechanism, which led to their dysfunction and eventual death. Furthermore, GABAergic interneuron loss causes dysregulation and hyperexcitability of neural networks in the hippocampus and cortex (Najm et al., 2019). APOE4 can induce abnormal excitability of neural networks in AD and epilepsy, suggesting that APOE4 may also be involved in mediating the mechanism of seizures in patients with AD.

GABAergic dysfunction may also contribute to seizures in AD. There is evidence of postsynaptic excitatory to inhibitory (E/I) imbalance in AD correlated with GABAergic dysfunction in humans (Lauterborn et al., 2021). A loss of GABA_A_ receptors was reported in AD patients’ brains (Calvo-Flores Guzman et al., 2018). The CA1 region of the hippocampus of AD patients also had a significant reduction in GABA_B_ receptor immunoreactivity (Iwakiri et al., 2005). In addition, GABAergic deficits and associated pathways seemed to be corrected by bumetanide. Bumetanide exposure was related to a significantly lower AD prevalence in individuals over age 65, and the treatment also rescued plasticity deficits and neuronal excitability in mice (Taubes et al., 2021). In a preclinical study, APP/PS1 transgenic mice exhibited a significant (50–60%) decrease in GABAergic neurons coexpressing somatostatin and neuropeptide cell loss (Ramos et al., 2006). Deficiencies in inhibitory GABAergic interneurons increase the excitability of neurons in many circuits by decreasing GABAergic inhibition (Wang et al., 2014). Moreover, a positive correlation existed between the global synaptic E/I ratio and neuronal firing (Iascone et al., 2020). Therefore, we cannot exclude the possibility that the synaptic E/I effect may be due to symptoms associated with AD, such as seizures (Amatniek et al., 2006).

## Seizure types in Alzheimer’s disease

Generalized tonic–clonic seizures (GTCS) have been reported as a common seizure type, but they are probably focal seizures with secondary generalization in AD patients (Larner, 2010). Increasing evidence suggests that focal seizures might be underestimated in patients with AD (Mendez and Lim, 2003). Focal non-motor seizures should be the predominant seizure type in patients with AD. The seizure semiology can include déjà vu, jamais vu, confusion, unexplained emotions (e.g., fear or euphoria), amnestic spells, speech arrest or sensory phenomena (e.g., metallic taste or epigastric rising sensation), which may overlap with other symptoms of AD (Vossel et al., 2017).

Transient epileptic amnesia (TEA), an unusual seizure type, sometimes occurs in patients with AD. Rabinowicz et al. (2000) first reported TEA in AD patients. The two patients transiently got lost in familiar environments caused by paroxysmal amnestic wandering and disorientation. A subsequent study reported that four AD patients suffered from TEA. Their acute and transient memory dysfunction showed a clear-cut response to antiseizure medications (ASMs) (Cretin et al., 2014). Additionally, some patients with AD may present with myoclonic seizure, with a higher risk in the advanced stages of disease (Asadollahi et al., 2019). New onset myoclonic jerks were observed in two cohorts of elderly patients with AD who developed from DS. EEG detected generalized spike-waves and polyspike-waves. Valproate, levetiracetam, topiramate and lamotrigine all appeared efficacious for myoclonic seizures in these patients (De Simone et al., 2010; Aller-Alvarez et al., 2017).

It is challenging to accurately identify seizure types in patients with AD only by clinical symptoms. Both AD patients and caregivers often rarely provide a reliable history. However, the episodes can be distinguished as epileptic events by their paroxysmal, recurrent and stereotyped nature, and further confirmed by epileptiform discharges on ictal EEG. LTM-VEEG and high-density EEG may be helpful for differential diagnosis (Vossel et al., 2017).

## Hazard of seizures and epileptiform discharges in Alzheimer’s disease

Seizures have adverse consequences on the natural course of AD and accelerate the decline of cognitive function in patients with AD. The results of a clinical retrospective study showed that 82% of patients with AD who suffered from seizures experienced sudden deterioration and required long-term care within 6 months of seizure onset. The study also revealed that approximately 50% of AD patients with seizures showed a significant decrease in language function compared to those without seizures (Volicer et al., 1995). In another study, patients with AD who had seizures began to experience a decline in cognitive function 5.5 years earlier than those without seizures (Vossel et al., 2013). In a recent study with similar results, the researchers also found that patients with AD who had seizures began to show a decline in cognitive function 3.6 years earlier than those without seizures (DiFrancesco et al., 2017). The presence of seizures in the memory clinic population predicted more severe impairments in activities of daily living (Baker et al., 2019). A preclinical study also showed that early seizure activity accelerated the depletion of hippocampal neural stem cells and impaired spatial discrimination in an AD model (Fu et al., 2019).

In addition, epileptiform discharges may also be associated with cognitive impairment. Hippocampal interictal epileptiform discharges might impair the maintenance and retrieval of memory (Kleen et al., 2013). Vossel et al. (2016) used the MMSE and executive function assessment and found that AD patients with subclinical epileptiform activity had a faster decline in overall cognitive ability than AD patients without. In a number of studies in AD model mice, epileptiform discharges have also been found to cause synaptic and cognitive impairment, which can be ameliorated by ASMs (Sanchez et al., 2012; Nygaard et al., 2015).

These findings suggest that both seizures and epileptiform discharges can cause cognitive impairment in patients with AD. Therefore, early and timely treatment may be necessary to prevent further deterioration of cognitive function in patients with AD.

## Treatment of seizures in Alzheimer’s disease

There is controversy about whether ASMs are needed in AD patients with seizures. Previously, Larner (2010) proposed that antiseizure treatment might not always be necessary because seizures were often non-convulsive and infrequent in patients with AD. Moreover, side-effects related to ASMs are of particular concern. There was a nationwide cohort of 70,718 patients with clinically verified AD from 2005 to 2011 in Finland. Among those patients, 3,058–5,769 patients used ASMs for epilepsy, neuropathic pain, psychiatric or depression disorders (Sarycheva et al., 2018b). The researchers conducted a series of studies about adverse events associated with ASMs in AD patients. They found that ASM users had an increased risk of stroke (37%) (Sarycheva et al., 2018a), pneumonia (92%) (Taipale et al., 2019), hip fracture (17%) (Pisa et al., 2021), and hospitalization (12 days) (Lavikainen et al., 2019) compared with non-users during the time follow-up. However, further subgroup analysis revealed different results. Newer ASM users had a lower risk of hip fracture than older ASMs, even non-users [(incidence rate (IR): 1.4 (1.1–1.9) vs. 2.6 (2.1–3.3) vs. 1.8 (1.6–1.9); Pisa et al., 2021]. Users with gabapentin and pregabalin had a lower hospitalization than valproate users (Lavikainen et al., 2019). In fact, fractures, pneumonia, hospitalization and cognitive decline may also be due to seizures or falls in AD patients. Cognitive adverse effects (CAEs) may be caused by some ASMs (phenobarbital, carbamazepine, phenytoin, valproate, primidone, barbexaclone, ethosuximide, clonazepam, zonisamide, and topiramate). Occasional use of ASMs with CAEs was associated with an increased risk of incident dementia (20%) and AD (16%), and regular use was associated with a greater risk (28 and 15%, respectively). However, adverse effects were not observed in the users of other ASMs (including levetiracetam, oxcarbazepine, lamotrigine, gabapentin, vigabatrin, pregabalin, tiagabine, lacosamide), regardless of whether the usage was occasional or regular (Taipale et al., 2018). On the other hand, clinical and basic studies have indicated that proper ASMs might potentially improve the prognosis of AD patients with epilepsy (Volicer et al., 1995; DiFrancesco et al., 2017; Liu et al., 2018). Levetiracetam might improve cognitive performance, specifically oral fluency items and attention, in AD patients with epilepsy (Cumbo and Ligori, 2010). Topiramate significantly restored nest-building and social interaction and reduced Aβ deposition in AD mice (Owona et al., 2019). Therefore, AD patients with recurrent seizures should take ASMs to prevent adverse consequences related to unpredictable episodes.

Age and drug interactions must also be considered when deciding to treat seizures. Most patients with AD are older and might be more prone to potential liver, kidney and heart toxicity. More importantly, due to their property to induce or inhibit cytochrome P450 enzymes (e.g., phenytoin, phenobarbital, carbamazepine and valproate), older ASMs may interact with concomitant medications. For example, some common medications of AD (donepezil, galantamine, rivastigmine, and ginkgo biloba) might inhibit hepatic enzymes and slow down the metabolism of ASMs, which may increase the risk of patient intoxication (Larner, 2010). In addition, sudden withdrawal of cholinesterase inhibitors might increase epileptic susceptibility and induce seizures in AD patients (Piecoro et al., 1998). Hence, older ASMs should problely be avoided in AD patients with epilepsy.

Currently, there is much evidence that levetiracetam and lamotrigine are used to control seizures in AD patients. A small-sample prospective observational study showed that levetiracetam (1,000 mg/d–2,000 mg/d) had an approximately 80% seizure-free rate for at least 1 year and a 14% withdrawal rate for adverse effects in late-stage AD patients with new onset epilepsy (Belcastro et al., 2007). For aMCI or AD patients with seizures, levetiracetam (250 mg/d–3,000 mg/d) and lamotrigine (50 mg/d–600 mg/d) exhibited better efficacy and tolerability than phenytoin (100 mg/d–600 mg/d) (Vossel et al., 2013). Similarly, compared with lamotrigine (25 mg/d–100 mg/d) and phenobarbital (50 mg/d–100 mg/d), levetiracetam (500 mg/d–2,000 mg/d) had fewer adverse events (28% for lamotrigine vs. 43.4% for phenobarbital and 17% for levetiracetam) in a prospective, randomized, case–control study of AD patients with epilepsy. At the 12-month visit, levetiracetam increased the MMSE score (−0.64 for lamotrigine vs. −1.57 for phenobarbital and + 0.23 for levetiracetam), and lamotrigine improved mood in the score for Cornell scale for depression (−0.72 for lamotrigine vs. +1.74 for phenobarbital and + 0.20 for levetiracetam) (Cumbo and Ligori, 2010). Furthermore, levetiracetam and lamotrigine have broad-spectrum efficacy in treating focal seizures, myoclonic seizures and generalized or unclassifiable seizures (Kanner and Bicchi, 2022). Lacosamide and eslicarbazepine are also prescribed ASMs in the setting of cognitive impairment, while limited data are available for their use in patients with AD. Considering the above reasons, levetiracetam and lamotrigine are recommended as current optimal therapeutic options for comorbid epilepsy in patients with AD (Cretin, 2018).

Given that Aβ and tau may be involved in epileptogenesis in AD, targeted therapy for Aβ or tau seems to be a rational choice for antiepilepsy therapy in AD. An animal experiment suggested that berberine restored Aβ-induced neurotoxicity and cognitive impairments (Haghani et al., 2015). Intracerebroventricular infusion of Aβ-specific antibody could prevent early-onset seizures in young ArcticAβ mice (Gschwind et al., 2018). Excitability of dentate gyrus granule cells in hippocampi from tau−/−, htau mice was significantly reduced with deletion of functional tau in an *in vitro* electrophysiological study (Cloyd et al., 2021). Another *in vivo* study showed that targeted reduction of tauopathy alleviated seizures and improved spatial learning and memory (Gao et al., 2020). Moreover, several clinical studies targeting Aβ- and tau-dependent pathways with immunotherapy are ongoing, but it is unclear whether therapeutic strategies can exhibit good antiepileptic efficacy (Liu and Wang, 2021).

Recently, the approaches deserving attention for treating epilepsy in patients with AD also include non-pharmacotherapeutic strategies, such as ketogenic diet therapies (KDTs), deep brain stimulation (DBS), and transcranial magnetic stimulation (TMS). KDTs were originally developed as a treatment for patients with intractable epilepsy, but studies have expanded their potential to treat other neurological disorders, such as AD, in recent years (Augustin et al., 2018; Koppel and Swerdlow, 2018). KDTs could improve cerebral blood flow and cognitive function in patients with AD (McDonald and Cervenka, 2019), which provides a potential for dietary management in AD patients with epilepsy. Additionally, chronic DBS might reduce neuronal loss and synaptic dysfunction in AD and epilepsy (McKinnon et al., 2019), and TMS could be used to improve cognitive function in dementia and to control seizures in epilepsy (Chen et al., 2008).

## Conclusion and prospects

Accumulating studies have revealed that seizures and epileptiform discharges are prevalent and persist throughout the course of AD, with a high probability of onset in the early stages. AD and epilepsy have a bidirectional connection. Epilepsy as a comorbidity of AD has been increasingly recognized, and a variety of potential mechanisms have been discovered. Various seizure types may occur in patients with AD, of which non-motor seizures are the most common. In addition, both seizures and epileptiform discharges are detrimental to the natural course of AD and may lead to a further decline in cognitive function in AD patients. Therefore, clinicians in the management of AD patients need to identify and intervene in seizures and epileptiform discharges early. At present, levetiracetam and lamotrigine are recommended as optimal ASMs for AD patients because of their preferable antiseizure efficacy and tolerability. Novel therapeutic strategies are worthy of attention. If the exact comorbidity mechanism of epilepsy in AD can be determined, relevant targeted treatment will substantially improve clinical outcomes.

## Author contributions

FY wrote and edited the manuscript. YH designed and revised the manuscript. All authors contributed to the article and approved the submitted version.

## Funding

This work was supported by the National Natural Science Foundation of China (Nos. 81260199, 81660228, and 82160261), Yunnan Province Talent Training Program (Nos. L-2019019 and H-2018056), Yunnan High-Level Talent Training Support Program Famous Doctor Special Project (No. RLMY20200005), and Project of Nanchong Science and Technology Bureau (No. 19SXHZ0051).

## Conflict of interest

The authors declare that the research was conducted in the absence of any commercial or financial relationships that could be construed as a potential conflict of interest.

## Publisher’s note

All claims expressed in this article are solely those of the authors and do not necessarily represent those of their affiliated organizations, or those of the publisher, the editors and the reviewers. Any product that may be evaluated in this article, or claim that may be made by its manufacturer, is not guaranteed or endorsed by the publisher.
